# Downregulation of Neurofilament Light Chain Expression in Human Neuronal-Glial Cell Co-Cultures by a Microbiome-Derived Lipopolysaccharide-Induced miRNA-30b-5p

**DOI:** 10.3389/fneur.2022.900048

**Published:** 2022-06-24

**Authors:** Aileen I. Pogue, Vivian R. Jaber, Nathan M. Sharfman, Yuhai Zhao, Walter J. Lukiw

**Affiliations:** ^1^Alchem Biotech Research, Toronto, ON, Canada; ^2^LSU Neuroscience Center, Louisiana State University Health Science Center, New Orleans, LA, United States; ^3^Department of Cell Biology and Anatomy, Louisiana State University Health Science Center, New Orleans, LA, United States; ^4^Department of Ophthalmology, Louisiana State University Health Science Center, New Orleans, LA, United States; ^5^Department Neurology, Louisiana State University Health Science Center, New Orleans, LA, United States

**Keywords:** neurofilament, microbiome and dysbiosis, lipopolysaccharide (endotoxin), Alzheimer's disease (AD), miRNA-30b-5p, neurofilament light (NF-L), NF-kB (p50/p65)

## Abstract

Microbiome-derived Gram-negative bacterial lipopolysaccharide (LPS) has been shown by multiple laboratories to reside within Alzheimer's disease (AD)-affected neocortical and hippocampal neurons. LPS and other pro-inflammatory stressors strongly induce a defined set of NF-kB (p50/p65)-sensitive human microRNAs, including a brain-enriched *Homo sapien* microRNA-30b-5p (hsa-miRNA-30b-5p; miRNA-30b). Here we provide evidence that this neuropathology-associated miRNA, known to be upregulated in AD brain and LPS-stressed human neuronal-glial (HNG) cells in primary culture targets the neurofilament light (NF-L) chain mRNA 3'-untranslated region (3'-UTR), which is conducive to the post-transcriptional downregulation of NF-L expression observed within both AD and LPS-treated HNG cells. A deficiency of NF-L is associated with consequent atrophy of the neuronal cytoskeleton and the disruption of synaptic organization. Interestingly, miRNA-30b has previously been shown to be highly expressed in amyloid-beta (Aβ) peptide-treated animal and cell models, and Aβ peptides promote LPS entry into neurons. Increased miRNA-30b expression induces neuronal injury, neuron loss, neuronal inflammation, impairment of synaptic transmission, and synaptic failure in neurodegenerative disease and transgenic murine models. This gut microbiota-derived LPS-NF-kB-miRNA-30b-NF-L pathological signaling network: **(i)** underscores a positive pathological link between the LPS of gastrointestinal (GI)-tract microbes and the inflammatory neuropathology, disordered cytoskeleton, and disrupted synaptic signaling of the AD brain and stressed brain cells; and **(ii)** is the first example of a microbiome-derived neurotoxic glycolipid having significant detrimental miRNA-30b-mediated actions on the expression of NF-L, an abundant neuron-specific filament protein known to be important in the maintenance of neuronal cell shape, axonal caliber, and synaptic homeostasis.

## Introduction

The gastrointestinal (GI) tract of *Homo sapiens* contains a complex, dynamic, and highly interactive community of microorganisms collectively known as the GI-tract microbiome possessing a staggering complexity and diversity. Composed of about ~10^**15**^ microorganisms from many thousands of different microbial species, the vast majority of human GI-tract microbes are composed of anaerobic or facultative anaerobic bacteria with aerobic bacteria, fungi, protozoa, *Archaebacteria* (an ancient intermediate microbial group between the prokaryotes and eukaryotes), viruses, and other microorganisms making up the remainder (1–3). Increasing research evidence has demonstrated that the composition of the GI-tract microbiome can significantly affect normal physiological homeostasis and contribute to the pathogenesis of diseases ranging from various types of inflammatory bowel disease to cancer to neurodegenerative disorders such as Alzheimer's disease [AD; (3–7)]. Gut microbiota can interact with the central nervous system (CNS) through the microbiota-gut-brain axis and through interactions mediated by metabolic and hormonal signaling, neural stimulation, and microbial secretions that both enhance and disrupt neurophysiology and neurological health. Deleterious microbial secretions are composed of neurotoxins, such as microbial amyloids, small bacterial RNAs, and endotoxins, such as *fragilysin* and lipopolysaccharide (LPS) that together represent some of the most pro-inflammatory and neurotoxic substances known (6–12). Together, complex mixtures of GI-tract-derived neurotoxins damage both colonic epithelial and neurovascular barriers, in part by inducing cleavage of the zonula adherens protein E-cadherin and other cell-cell adhesion molecules, thereby disrupting cell-cell adhesion, and enabling the translocation of these potent neurotoxins across aged or damaged plasma membranes, and into the systemic circulation, into CNS and PNS compartments and across the plasma membrane of brain cells (3–12). One major class of microbiome-derived neurotoxin is the Gram-negative bacteria-derived lipoprotein glycoconjugate lipopolysaccharide (LPS) that has been reported by several independent research groups to reside within the brain cells and CNS tissues of aged patients affected with AD and in AD murine models (10–15). Many different variations of LPS are derived from different human microbiome-resident Gram-negative bacteria; for example, species such as the anaerobic bacterium *Bacteroides fragilis* are capable of secreting particularly pro-inflammatory and neurotoxic forms of LPS, such as BF-LPS, which penetrate physiological barriers, including brain cell plasma membranes (8–18). Importantly, Aβ peptides, one neuropathological hallmark for AD, have recently been shown to further support the translocation of LPS into neurons, probably *via* transient channel formation through the neuronal plasma membrane (6–12).

This “Perspectives” paper ties together several recent observations linking increased LPS and LPS-induced NF-kB signaling with increases in a pathogenic human CNS-enriched NF-kB-sensitive microRNA-30b. We provide the first evidence that increased miRNA-30b is capable of targeting the 3'-UTR of the neuron-specific neurofilament light (NF-L) chain messenger RNA (mRNA), thus linking this action to the decreased expression of NF-L, a cytoskeletal element known to be downregulated within CNS neurons in AD affected brain, in stressed HNG cells in primary culture and in transgenic murine models of AD (14–21). In doing so, NF-L depletion disrupts normal neuronal cell shape, cytoarchitecture, and synaptic organization. This is the first example of a microbiome-initiated pathogenic pathway linking LPS, an abundant microbial glycolipid neurotoxin, with the miRNA-mediated downregulation of an essential neuron-specific cytoskeletal component normally required to maintain the cytoarchitecture and signaling functions of the neuron.

## Neurofilament Light Chain Protein and AD

The neuron-specific neurofilament light (NF-L) chain protein of the neurofilament (NF) triplet bundle consisting of NF-L, neurofilament medium, and heavy chains (NF-M, NF-H): **(i)** is normally the most abundant neurofilamentous structural element in neurons; **(ii)** is a key scaffolding component of the axoskeleton of healthy neurons, to which other neuronal cytoskeletal proteins attach; and **(iii)** interacts directly with multiple synaptic-phosphoproteins to support and coordinate neuronal cell shape, cytoarchitecture, neurotransmission, synaptogenesis, and inter-neuronal synaptic signaling (19–24). A remarkably high number of neurological disorders exhibit NF-L degradation and the liberation of NF-L from neuron-specific compartments, mobilization, and enrichment into pathological biofluids in the periphery; this may be due to deficits in plasma membrane barrier's integrity and pathological transport and/or vesicle-mediated trafficking dysfunction of this highly stable 61,517 Da filament protein from diseased neurons [(19–28); https://www.genecards.org/cgi-bin/carddisp.pl?gene=NEFL; last accessed 24 April 2022]. Originally thought to be a specific blood-borne biomarker for AD, NF-L abundance in the CSF and other circulating biofluids has more recently been considered an easily quantifiable and promising peripheral biomarker for all-cause neurodegeneration in both clinical and research settings (20–26). Although NF-L may not be a disease-specific peripheral biomarker, its presence in the blood and CSF has aided in the early detection, diagnosis, prognosis, and prediction of time-to-symptom onset in all-cause dementia, including frontotemporal dementia (FTD), amyotrophic lateral sclerosis (ALS), Huntington's disease, Parkinson's disease (PD), human prion disease (PrD) and AD (20–25). It is somewhat paradoxical that NF-L abundance is increased in peripheral biofluids in multiple forms of neurodegeneration while being significantly downregulated within CNS neurons, however molecular-genetic mechanisms involving altered NF-L trafficking in AD and other neurodegenerative diseases and the utilization of cellular exosomes (EXs), extracellular microvesicles (EMVs) and other altered translocation mechanisms for NF-L have recently been proposed to clarify this perplexing observation (18, 20–23).

Within neocortical neurons of the degenerating AD brain is observed a significant loss of NF-L mRNA and protein that cannot be explained by neuronal loss alone (26–28). Decreased NF-L abundance is also observed in other forms of both acute and chronic neuronal injury and LPS-stressed human neuronal-glial (HNG) cells in primary co-culture (18–22, 27, 28). NFs are highly critical and stable scaffolding components of the axoskeleton of healthy neurons interacting directly with multiple synaptic phosphoproteins to support and coordinate neuronal cell shape, cytoarchitecture, synaptogenesis, and neurotransmission (18–23). In multiple forms of human age-related neurological disease are observed a pathological shift of NF-L from an intracellular neuronal cytoplasmic location into various biofluid compartments, and NF-L is currently categorized as a peripheral biomarker for the diagnosis, prognosis, time-to-symptom, and response-to-drug-treatment of all-cause dementia (18–26). Downregulated NF-L within neurons strongly correlates with the observed axonal and neuronal atrophy, neurite deterioration, reduction in axonal caliber, and synaptic disorganization in tissues affected by AD and other progressive and age-related neurological diseases, but the molecular-genetic mechanism for decreased NF-L abundance has, up until now, not been explored. Dysregulated brain-abundant microRNA abundance, speciation, and complexity have been strongly implicated in the molecular-genetic mechanism of AD and other forms of progressive neurodegeneration of the human brain and CNS (29–33). Recent evidence continues to support the idea of a human brain-abundant pathology-associated miRNA-30b in various disease states and the targeting of the NF-L mRNA 3'-UTR that may account, in part, for this decreased output of NF-L mRNA, protein, and expression in LPS-stressed neurons, and have relevance to the altered neuronal signaling capabilities characteristic of AD-affected neurons.

## miRNAs Under NF-kB-Regulation

The term “microRNA” (miRNA) denotes a species of ~22 nucleotide (nt), small non-coding RNA (sncRNA) that, *via* base-pair complementarity, recognizes and binds to target messenger RNAs (mRNAs) to shape the transcriptome of the cell (33–39). The major mode of action of miRNAs is accomplished by the recognition and binding of these sncRNAs to the 3'-untranslated region (3'-UTR) of their target mRNAs, and by inhibiting the expression of genetic information encoded by that mRNA negatively regulates the posttranscriptional expression of genes (33–36). This miRNA-mRNA regulatory and modulatory system is highly complex and interactive as different miRNAs can target a single mRNA, and single mRNAs may be targeted by more the one miRNA (32–36). Interestingly, the total number of miRNAs in *Homo sapiens* currently numbers about ~2,650, although the number of abundant and easily detected miRNAs in the human brain and CNS only numbers about 45–50, many of which are under NF-kB regulatory control [(33–40); https://lcsciences.com/services/microarray-services/mirna/; last accessed 24 April 2022].

A small subset of NF-kB-regulated miRNAs has been identified and characterized in the AD neocortex and hippocampal CA1 region, and in reactive-oxygen species (ROS)-, cytokine interleukin 1-beta (IL-1β), amyloid-beta 42 (Aβ42) peptide, and/or lipopolysaccharide-(LPS) stressed human neuronal-glial (HNG) cells in primary co-culture (36–49). Overall these findings suggest that the upregulation of this same small miRNA family orchestrates a pro-inflammatory and pathogenic gene expression program, which may explain many of the pathological aspects of AD onset and propagation including: **(i)** the failure of the microglial-mediated clearance of end-stage peptides from brain cells and amyloidogenesis; and **(ii)** a significant downregulation in the production of essential cytoskeletal components and synaptic signaling elements.

## miRNA-30b and Neurodegeneration

The NF-kB regulated miRNA-30b is a brain-enriched member of the miRNA-30 gene family (41–45). The expression of miRNA-30b is implicated in playing a crucial homeostatic regulatory role in tissue and organ development and the pathogenesis of an array of diseases from cancer to progressive inflammatory neurodegenerative disorders, such as AD (41–46). Multiple independent reports indicate that the NF-kB-inducible miRNA-30b: **(i)** is upregulated in AD and animal models of AD (43–46); **(ii)** that the overexpression of miRNA-30b in the hippocampus impairs basal synaptic transmission, long term potentiation (LTP), learning, and memory and is associated with a significant reduction in dendritic spine density (42, 43); **(iii)** causes synaptic and cognitive dysfunction in AD and in AD animal models (https://www.ncbi.nlm.nih.gov/gene/407030; 2022; last accessed 24 April 2022; 42,44); **(iv)** is significantly upregulated by lipopolysaccharide (LPS) or protozoan-mediated infection of human epithelial cells (46); and **(v)** targets the 3'-UTR of the mRNA encoding sirtuin 1 (SIRT1), a ubiquitous deacetylase that regulates numerous cellular functions at the level of gene expression, including aging, lipid homeostasis, and inflammatory signaling (48). Because of the abundance of this NF-kB-upregulated miRNA-30b in the human brain and CNS neurons, with its significant over-expression in AD and this miRNA's known impact on human neurophysiological effects and pathways relevant to neurodegenerative disease, we further examined miRNA-30b as a potential regulator of NF-L gene expression.

## Recent Studies on miRNA-30b-NF-L mRNA Interaction

**Figure 1:**
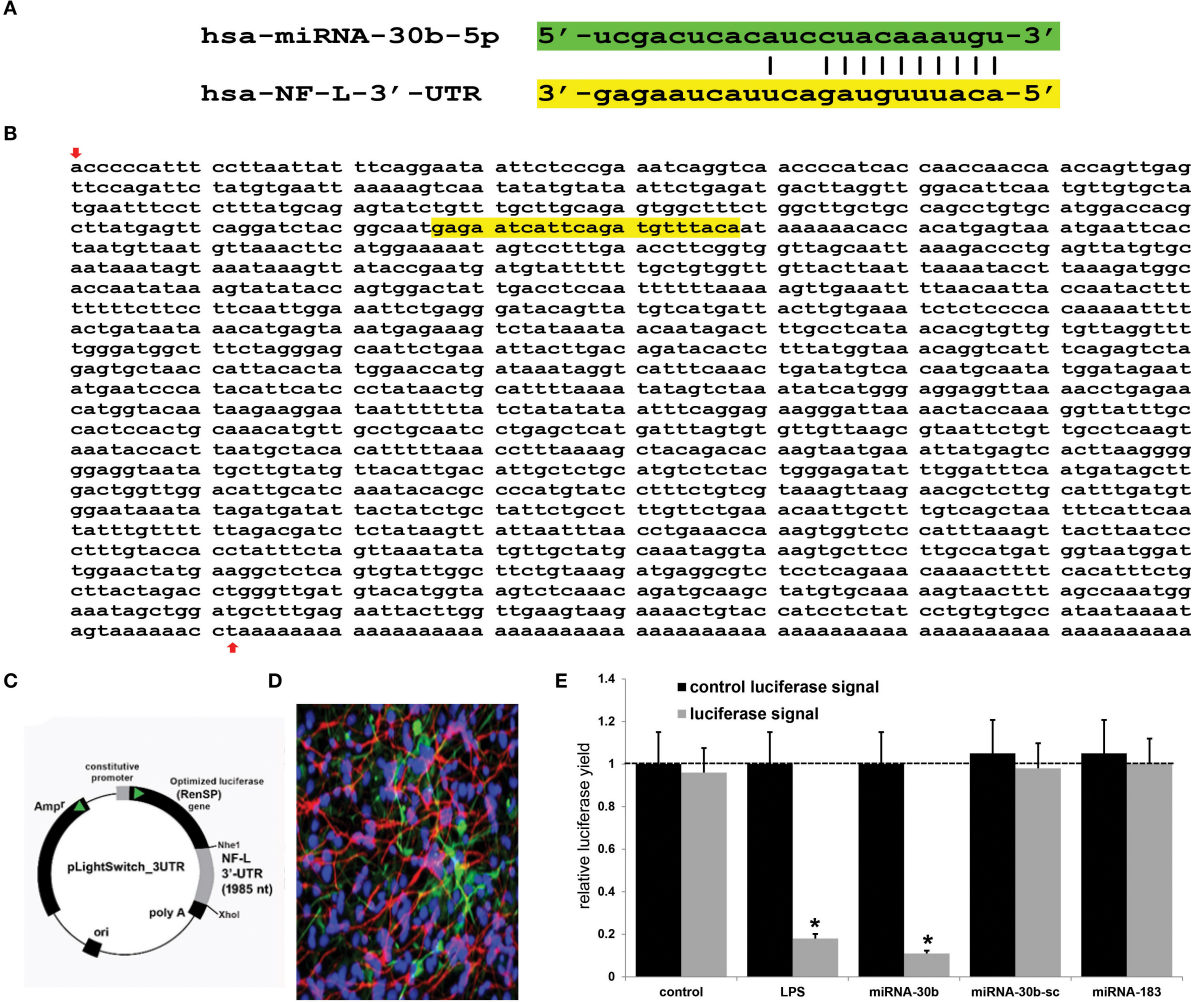
Analysis of the hsa-miRNA-30b-5p (miRNA-30b) interaction with the *Homo sapien* NF-L 3'-UTR; **(A)** representation of the nucleotide complementarity between the 22 nucleotide (nt) hsa-miR-30b-5p (highlighted in green; encoded at the miRNA-30 gene cluster on human chromosome (chr) 8q24.22; https://www.genecards.org/cgi-bin/carddisp.pl?gene=MIR30B) and nt position 266–287 of the NF-L mRNA 3'-UTR non-coding region (highlighted in yellow; encoded at human chr 8p21.1; https://www.genecards.org/cgi-bin/carddisp.pl?gene=NEFL); the microRNA target prediction database (miRDB; http://mirdb.org/cgi-bin/targetdetail.cgi?targetID=2099169; last accessed April 24, 2022) for miRNA-30b and NF-L (NEFL; NCBI Gene ID 4747; GenBank Accession NM_006158) indicates a very high miRNA-mRNA target score of 84 and a strong 10 nt ‘seed' sequence location at 278-287 nt of the NF-L 3'-UTR [see also “**(B)**” below]; **(B)** the NF-L 3'-UTR gene sequence; the inverted red arrow indicates the start of the NF-L 3'-UTR non-coding sequence; last upward pointing red arrow is the end of the NF-L 3'-UTR; note that additional adenosine groups are present in the mature NF-L mRNA (and 3' end of the NF-L 3'-UTR); **(C)** the NF-L-mRNA-3′-UTR expression vector luciferase reporter assay (pLight Switch-3′UTR; Cat#S810535; Switchgear Genomics, Palo Alto CA); in this vector, the entire 1,985 nucleotide NF-L 3′-UTR was ligated into the unique Nhe1-Xho1site; not drawn to scale; **(D)** human neuronal-glial (HNG) cells, 2 weeks in primary culture; neurons (red stain; λmax = 690 nm), DAPI (blue nuclear stain; λmax = 470 nm) and glial fibrillary associated protein (GFAP; glial-specific green stain; λmax = 520 nm); the HNG cell culture is about 60% confluent and at 2 weeks of culture contains about 70% neurons and 30% astroglia (7, 14, 18, 39, 40, 49); human neurons do not culture well in the absence of glia; neurons also show both extensive cytoarchitecture and display electrical activity (unpublished; Lonza Research and Development, Walkersville MD, USA); 40X magnification; HNG cells transfected with the NF-L-mRNA-3′-UTR expression vector luciferase reporter were treated exogenously with LPS (20 ng/ml cell culture medium, 48 hr), a stabilized miRNA-30b, a scrambled control miRNA-30b (miRNA-30b-sc) or control miRNA-183; see (14, 18, 39, 49) and text for further details on all reagents and methods used in these experiments; **(E)** compared to control, HNG cells transfected with a scrambled (sc) control pLightSwitch-3'-UTR vector, the NF-L-mRNA-3′-UTR vector exhibited decreased luciferase signal to a mean of 0.18-fold of controls in the presence of exogenous LPS and 0.11 in the presence of miRNA-30b; this same vector exhibited no change in relative luciferase yield in the presence of a control miRNA-30b-sc or miRNA-183; for each experiment (using different batches of HNG cells) a control luciferase signal was generated that included separate controls with each analysis; in addition a control vector β-actin-3′-UTR showed no significant effects on the relative luciferase signal yield after treatment with either miRNA-183 or miRNA-30b (data not shown); a dashed horizontal line set to 1 is included for ease of comparison; *N* = 5; *p < 0.01 (ANOVA); values represent mean +/- 1 standard deviation (S.D.); Microsoft Excel Analysis ToolPak, Excel for Microsoft 365; https://support.microsoft.com/en-us/office/use-the-analysis-toolpak-to-perform-complex-data-analysis-6c67ccf0-f4a9-487c-8dec-bdb5a2cefab6. The results suggest a physiologically relevant miRNA-30b-NF-L-mRNA-3′-UTR interaction and a miRNA-30b-mediated downregulation of NF-L expression in HNG cells. This pathogenic interaction may be related to the downregulation of other immune, inflammatory, and synaptic system genes by upregulated miRNAs in the CNS resulting in a deficit in cytoskeletal and synaptic organization and trans-synaptic signaling (7, 21, 26, 27, 31, 32, 36, 38–40, 50).

Using miRBase (mirbase.org Release 22.1) and the miRDB database search engine (http://mirdb.org/cgi-bin/search.cgi; last accessed 24 April 2022), it was predicted that the single copy the human neurofilament light chain gene [NF-L; NEFL, NeFL; gene 4747; 5767 base pairs (bp) located at human chr 8p21.2; accession number MIMAT0000420; https://www.genecards.org/cgi-bin/carddisp.pl?gene=NEFL; last accessed 24 April 2022] encodes a 3,584 nucleotide (nt) A+T-rich linear mRNA that possesses a 1,985 nt 3'-UTR (NCBI Reference Sequence: NM_006158.5; Ensembl:ENSG00000277586MIM:162280; AllianceGenome:HGNC:7739; https://www.ncbi.nlm.nih.gov/gene?Db=gene&Cmd=DetailsSearch&Term=4747; last accessed 24 April 2022). The NF-L 3'-UTR region has the potential to be targeted by at least 124 different miRNAs (Figure 1 and Supplementary Table 1). Because of multiple previous studies verifying its brain involvement on CNS pathology, the brain-enriched NF-kB-sensitive miRNA-30b and NF-L 3'-UTR interaction was studied further (44–48). To validate a functional miRNA-30b-NF-L 3'-UTR interaction, we used HNG cells (at 2 weeks in culture) transfected with a miRNA-30b-NF-L 3'-UTR expression vector luciferase reporter assay (pLightSwitch-3′UTR; Cat#S810535; Switchgear Genomics, Palo Alto CA). In this vector, the entire 1,985 nucleotides' NF-L 3′-UTR had been ligated into the unique Nhe1-Xho1site; all experimental procedures and the use of pLightSwitch-3′UTR luciferase-reporter vectors have been previously described in detail (18, 39, 49). HNG cells were subsequently treated with a stabilized miRNA-30b, a scrambled control miRNA-30b (miRNA-30b-sc), a control miRNA-183 or LPS (EC No: 297-473-0; MDL No: MFCD00164401; Cat No: L4391; Millipore Sigma. St Louis MO, USA) at 20 ng/ml cell culture medium for 48 hr as previously described [(18, 39, 49); Figures 1C,E]. Compared to controls, HNG cells transfected with the NF-L-mRNA-3′-UTR vector exhibited decreased luciferase signal to a mean of 0.18-fold of controls in the presence of exogenous LPS (20 ng/ml of HNG cell culture medium), and a mean of 0.11 in the presence of miRNA-30b; this same vector exhibited no significant change in luciferase signal yield in the presence of the control sncRNAs miRNA-30b-sc or miRNA-183. In addition, a control vector β-actin-3′-UTR showed no significant effects on the relative luciferase signal yield after treatment with either miRNA-30b or miRNA-183 (data not shown). Taken together, these results suggest a physiologically relevant miRNA-30b-NF-L-mRNA-3′-UTR interaction conducive to the verification of a miRNA-30b-mediated downregulation of NF-L expression in HNG cells. This NF-kB-sensitive miRNA-30b-mediated pathogenic interaction may be related to the downregulation of other immune, inflammatory, and synaptic system gene expression by pathological upregulation of miRNAs in the CNS, thereby resulting in altered cytoskeletal dynamics and neuronal atrophy as is observed in AD brain and in AD cellular and animal models associated with the progressive development of neocortical pathology (18, 33, 37–40, 42, 46–49, 51).

## Discussion

The human GI-tract microbiome is a rich and dynamic source of microorganisms of staggering diversity and complexity. GI-tract commensal microbes are generally beneficial to global human metabolism, immunity, and health. However, enterotoxigenic forms of these same microbes possess significant potential to secrete some of the most neurotoxic and pro-inflammatory biopolymers known. These neurotoxins have been found to significantly disrupt normal gene expression patterns in the CNS. These include multiple species of Gram-negative bacteria-derived neurotoxic-glycolipids, such as LPS, long known to be an inducer of pro-inflammatory, and altered immunological signaling in infection and human disease (14–18, 47–50, 52, 53). It should be mentioned that although there has been observed a significant variability in microbial abundance, speciation and complexity even amongst healthy individuals and that it has been difficult to link specific microbial abundance patterns with any neurological disease, certain GI-tract microbial compositions appear to be more conducive to the production of secreted pathological neurotoxins that include LPS (1, 2, 47–50, 52, 53). It has also been appreciated for some time that the toxins that include LPS drive pathological pro-inflammatory signaling programs in neurons in large part *via* the induction of NF-kB and the upregulation of NF-kB-sensitive miRNAs. However, the details of the molecular-genetic mechanisms and signaling pathways involved still require a more thorough investigation (4, 40, 46, 47, 50, 52–54).

In this Perspectives paper, from recent experiments from our laboratory and multiple current research reports in the last several years, we have integrated data and provided evidence of microbial-derived LPS-mediated induction of NF-kB and miRNA-30b, whose upregulation appears to target and downregulate expression of the NF-L-3'-UTR whose mRNA encodes a critical neuron-specific component of the neuronal cytoskeleton and cytoarchitecture. Previously, microbial-derived LPS has been shown to induce NF-kB and NF-kB-sensitive miRNA-30b signaling (42, 45–47) and pathologically miRNA-30b is robustly upregulated in the brains of both patients with AD and in Aβ-peptide over-expressing transgenic murine models of AD (TgAD), while expression of its multiple mRNA targets that maintain neuronal structure and synaptic signaling, such as the NF-L transcript, is significantly downregulated (42, 45–48). We provide molecular-genetic evidence that LPS and miRNA-30b in HNG cells in primary culture both target the NF-L 3'-UTR, a process known to ultimately result in NF-L downregulation. It is of further interest: **(i)** that the overexpression of miRNA-30b in the hippocampus of normal wild-type mice has been reported to impair synaptic and cognitive functions, mimicking those seen in TgAD models; **(ii)** that, conversely, knockdown of endogenous miRNA-30b in murine models prevents synaptic and cognitive decline; **(iii)** that the expression of miRNA-30b is significantly upregulated by pro-inflammatory cytokines and Aβ peptides through NF-κB signaling; **(iv)** that miRNA-30b, upregulated in the brains of patients with AD has been found to impair synaptic transmission, consequently leading to progressive synaptic failure and, thus, promoting AD development; and **(v)** that miRNA-30b over-expression induces neuronal injury, neuron loss, and proliferates specific biomarkers for neuronal inflammation (41–46). While both miRNA-30b and NF-L are encoded on the same human chromosome 8, the significance of this, if any, is currently not yet understood (see legend to Figure 1).

**Figure 2:**
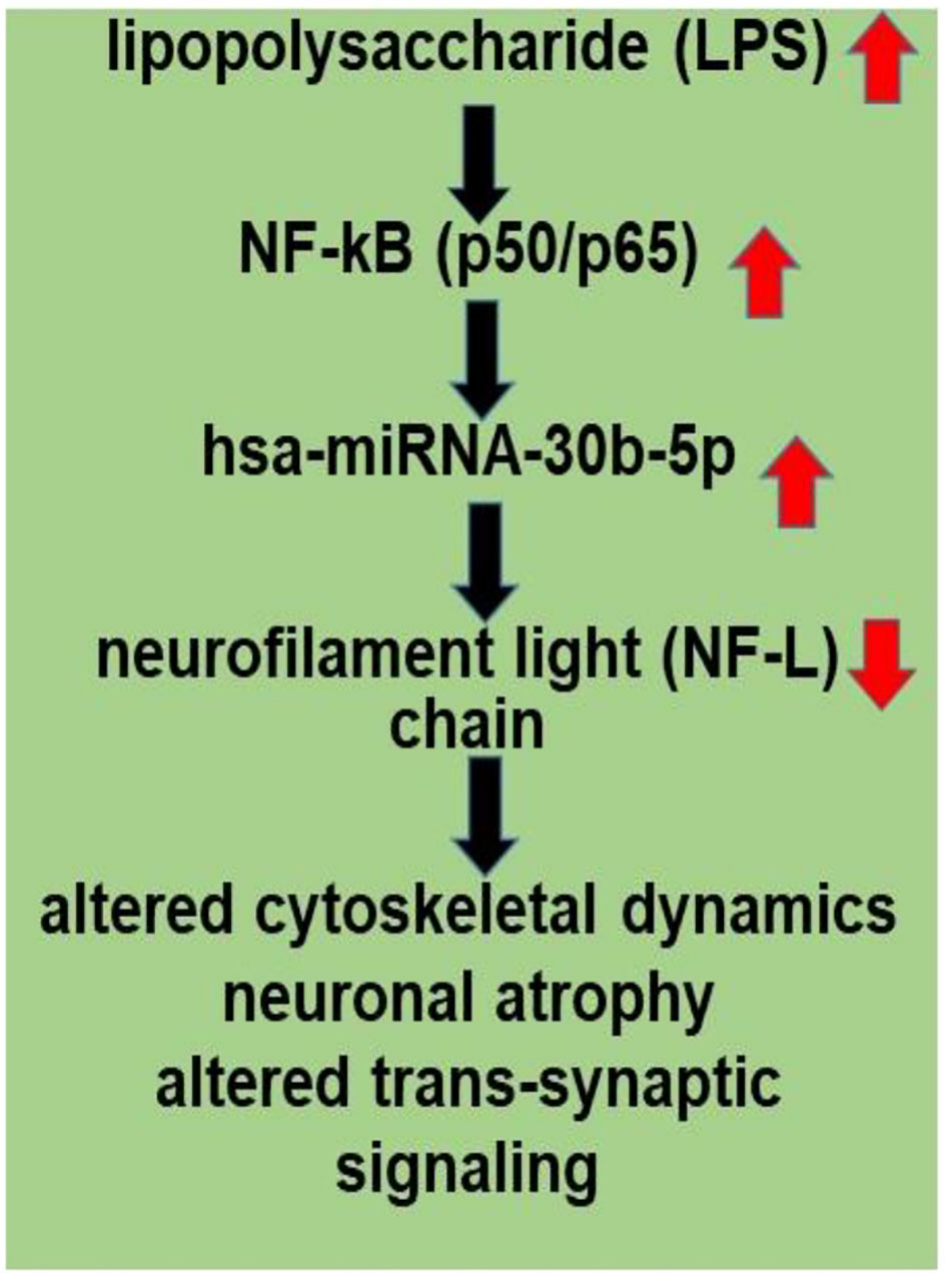
LPS, present in brain cells affected with AD, has an inhibitory effect on NF-L expression; a human microbiome-derived lipopolysaccharide (LPS)-NF-kB-miRNA-30b-NF-L pathological signaling pathway may be in part responsible for driving altered cytoskeletal dynamics, neuronal atrophy and altered trans-synaptic signaling in stressed human neuronal-glial (HNG) cells in primary culture and in Alzheimer's disease (AD) brain. LPSs are neurotoxic glycolipids derived from the outer cell wall of non-capsulated Gram-negative bacteria; normally they contribute to the integrity of the outer cell wall membrane and protect the cell against the action of bile salts and lipophilic antibiotics (50, 52, 53). Both microbial infection and LPS are strong inducers of NF-kB signaling in neurons and other human cell types. miRNA-30b is under transcriptional control by NF-kB, and the neuron-specific NF-L chain mRNA-3'-UTR is a target for miRNA-30b. Other miRNAs may be involved (see Supplementary File 1). Disruption and insufficiency of NF-L abundance within the neuron are in part responsible for disturbances in neuronal cytoarchitecture, atrophy, and synaptic aberrations as is observed in stressed human brain cells and in AD-affected neocortex.

An improved understanding of the interaction between the GI tract-CNS axis and the GI-tract microbiome and AD has considerable potential to lead to new diagnostic and therapeutic strategies in the clinical management of AD and other lethal, progressive, and age-related neurodegenerative disorders. Current findings further support the hypothesis of an altered miRNA-mRNA coupled signaling network in AD, much of which is supported by recently described experimental findings in the scientific literature. Targeting and modulating GI-tract microbiome LPS-mediated miRNA-30b-regulated NF-L pathways and other miRNA-mediated gene expression circuitry should be valuable in the design of future therapeutic strategies (Figure 2). The overall goals of these strategies are that the support and maintenance of cytoskeletal structures essential for synaptic plasticity may more effectively manage the many neurological diseases in which NF-L gene expression and abundance play a determinant and defining role. Lastly, dietary-based modifications of microbial dysbiosis may be an attractive means to modify the abundance, speciation, and complexity of enterotoxigenic forms of AD-relevant microbes and their potential for the pathological discharge of highly neurotoxic microbial-derived secretions that include LPS (4, 50, 53–57).

## Data Availability Statement

The original contributions presented in the study are included in the article/Supplementary Material, further inquiries can be directed to the corresponding author.

## Ethics Statement

Human neuronal-glial (HNG) cells were obtained from commercial sources (Lonza Biosciences, Muenchensteinerstrasse 38, CH-4002 Basel, Switzerland). The culture of HNG cells, acquisition and handling procedures were carried out in accordance with the ethics review board policies at both the donor institutions and at the Louisiana State University Health Sciences Center (LSU-HSC) New Orleans. The work in this study was approved by the IACUC protocols #3726 and IBC #18059 at the LSU Health Sciences Center, New Orleans LA 70112 USA.

## Author Contributions

AP, VJ, NS, YZ, and WL collected, analyzed, and summarized the current NF-L (NEFL) and miRNA-30b literature. AP, VJ, YZ, and WL performed the experiments and data extraction. WL wrote the article. All authors contributed to the article and approved the submitted version.

## Funding

Research on miRNA and miRNA-mRNA interactions in the Lukiw laboratory, involving the cytoskeleton of neurons and synaptic signaling, AD innate-immune response, and neuro-inflammation, was supported through Translational Research Initiative Grant from LSUHSC, the Brown Foundation, Joe and Dorothy Dorsett Innovation in Science Healthy Aging Award, and NIA Grants AG18031 and AG038834.

## Author Disclaimer

The content of this manuscript is solely the responsibility of the authors and does not necessarily represent the official views of the NIH.

## Conflict of Interest

Alchem Biotech Research is a non-profit, non-commercial research, bioinformatics, and statistical analysis facility. The authors declare that the research was conducted in the absence of any commercial or financial relationships that could be construed as a potential conflict of interest.

## Publisher's Note

All claims expressed in this article are solely those of the authors and do not necessarily represent those of their affiliated organizations, or those of the publisher, the editors and the reviewers. Any product that may be evaluated in this article, or claim that may be made by its manufacturer, is not guaranteed or endorsed by the publisher.
