# Supplementary material for: Downregulation of Neurofilament Light Chain Expression in Human Neuronal-Glial Cell Co-Cultures by a Microbiome-Derived Lipopolysaccharide-Induced miRNA-30b-5p
Source: Front Neurol. 2022 Jun 24;13:900048. doi: 10.3389/fneur.2022.900048 (PMC9263091; doi:10.3389/fneur.2022.900048)
Supplement: Supplementary file 1 [file Data_Sheet_1.pdf]

Supplementary File 1

The 3' untranslated region (3'-UTR) of the human neurofilament light (NF-L; NEFL; NeFL; gene 4747) chain gene encoded at human chr8p21.2 is predicted to be targeted by ~124 miRNAs using the miRNA Database search engine (an online database for miRNA target prediction and functional annotations; all the targets in miRDB were predicted by this bioinformatics tool; http://mirdb.org/cgi-bin/search.cgi; for more details see http://mirdb.org/cgi-bin/search.cgi; last accessed 25 May 2022); hsa-miR-30b-5p (Gene ID: 407030; encoded at chr 8q24.22) ranks 24th on this list with a target score of 84; interestingly all 5 members of the human miRNA-30-5p gene family (Target Rank 22-26) have potential targets in the NF-L 3'-UTR; the complementarity of hsa-miR-30b-5p to its NF-L 3'-UTR target is 100% homologous to a 10 nt 'seed sequence' region located within the 3' end of the human miRNA-30b (see **Figure 1A and 1B**); other miRNAs may participate in targeting the NF-L 3'-UTR and/or other NF-L mRNA regions and participate in the modulation or down-regulation of NF-L expression.

Target Rank	Target Score	miRNA Name	Gene Symbol	Gene Description
1	99	hsa-miR-3140-5p	NF-L, NEFL	neurofilament light
2	97	hsa-miR-4699-3p	NF-L, NEFL	neurofilament light
3	94	hsa-miR-32-5p	NF-L, NEFL	neurofilament light
4	94	hsa-miR-92b-3p	NF-L, NEFL	neurofilament light
5	94	hsa-miR-92a-3p	NF-L, NEFL	neurofilament light
6	93	hsa-miR-585-5p	NF-L, NEFL	neurofilament light
7	93	hsa-miR-5003-5p	NF-L, NEFL	neurofilament light
8	92	hsa-miR-208b-5p	NF-L, NEFL	neurofilament light
9	92	hsa-miR-367-3p	NF-L, NEFL	neurofilament light
10	92	hsa-miR-363-3p	NF-L, NEFL	neurofilament light
11	92	hsa-miR-208a-5p	NF-L, NEFL	neurofilament light
12	92	hsa-miR-25-3p	NF-L, NEFL	neurofilament light
13	91	hsa-miR-3143	NF-L, NEFL	neurofilament light
14	89	hsa-miR-302c-5p	NF-L, NEFL	neurofilament light
15	88	hsa-miR-330-3p	NF-L, NEFL	neurofilament light
16	87	hsa-miR-190a-3p	NF-L, NEFL	neurofilament light
17	86	hsa-miR-6796-5p	NF-L, NEFL	neurofilament light
18	86	hsa-miR-4330	NF-L, NEFL	neurofilament light
19	86	hsa-miR-507	NF-L, NEFL	neurofilament light
20	85	hsa-miR-183-5p	NF-L, NEFL	neurofilament light
21	85	hsa-miR-708-3p	NF-L, NEFL	neurofilament light
22	84	hsa-miR-30a-5p	NF-L, NEFL	neurofilament light
23	84	hsa-miR-30c-5p	NF-L, NEFL	neurofilament light
24	84	hsa-miR-30b-5p	NF-L, NEFL	neurofilament light
25	84	hsa-miR-30d-5p	NF-L, NEFL	neurofilament light
26	84	hsa-miR-30e-5p	NF-L, NEFL	neurofilament light
27	83	hsa-miR-583	NF-L, NEFL	neurofilament light
28	83	hsa-miR-4714-5p	NF-L, NEFL	neurofilament light
29	82	hsa-miR-23a-3p	NF-L, NEFL	neurofilament light
30	82	hsa-miR-514a-5p	NF-L, NEFL	neurofilament light
31	82	hsa-miR-23c	NF-L, NEFL	neurofilament light
32	82	hsa-miR-23b-3p	NF-L, NEFL	neurofilament light
33	82	hsa-miR-12135	NF-L, NEFL	neurofilament light
34	82	hsa-miR-7702	NF-L, NEFL	neurofilament light
35	81	hsa-miR-545-5p	NF-L, NEFL	neurofilament light
36	80	hsa-miR-3661	NF-L, NEFL	neurofilament light
37	80	hsa-miR-557	NF-L, NEFL	neurofilament light

38	80	hsa-miR-92a-1-5p	NF-L, NEFL	neurofilament light
39	79	hsa-miR-4282	NF-L, NEFL	neurofilament light
40	79	hsa-miR-6746-5p	NF-L, NEFL	neurofilament light
41	79	hsa-miR-5696	NF-L, NEFL	neurofilament light
42	78	hsa-miR-3664-5p	NF-L, NEFL	neurofilament light
43	78	hsa-miR-6771-5p	NF-L, NEFL	neurofilament light
44	77	hsa-miR-4760-3p	NF-L, NEFL	neurofilament light
45	76	hsa-miR-631	NF-L, NEFL	neurofilament light
46	76	hsa-miR-196a-3p	NF-L, NEFL	neurofilament light
47	75	hsa-miR-150-3p	NF-L, NEFL	neurofilament light
48	74	hsa-miR-192-5p	NF-L, NEFL	neurofilament light
49	74	hsa-miR-3714	NF-L, NEFL	neurofilament light
50	74	hsa-miR-215-5p	NF-L, NEFL	neurofilament light
51	73	hsa-miR-3137	NF-L, NEFL	neurofilament light
52	73	hsa-miR-8066	NF-L, NEFL	neurofilament light
53	72	hsa-miR-329-5p	NF-L, NEFL	neurofilament light
54	72	hsa-miR-3125	NF-L, NEFL	neurofilament light
55	71	hsa-miR-6868-3p	NF-L, NEFL	neurofilament light
56	71	hsa-miR-3606-5p	NF-L, NEFL	neurofilament light
57	71	hsa-miR-545-3p	NF-L, NEFL	neurofilament light
58	71	hsa-miR-218-1-3p	NF-L, NEFL	neurofilament light
59	71	hsa-miR-12136	NF-L, NEFL	neurofilament light
60	70	hsa-miR-1248	NF-L, NEFL	neurofilament light
61	70	hsa-miR-6859-5p	NF-L, NEFL	neurofilament light
62	70	hsa-miR-3175	NF-L, NEFL	neurofilament light
63	70	hsa-miR-6757-5p	NF-L, NEFL	neurofilament light
64	70	hsa-miR-3916	NF-L, NEFL	neurofilament light
65	69	hsa-miR-140-3p	NF-L, NEFL	neurofilament light
66	69	hsa-miR-29b-2-5p	NF-L, NEFL	neurofilament light
67	68	hsa-miR-193a-5p	NF-L, NEFL	neurofilament light
68	66	hsa-miR-4539	NF-L, NEFL	neurofilament light
69	66	hsa-miR-587	NF-L, NEFL	neurofilament light
70	66	hsa-miR-12113	NF-L, NEFL	neurofilament light
71	66	hsa-miR-513b-5p	NF-L, NEFL	neurofilament light
72	64	hsa-miR-7-1-3p	NF-L, NEFL	neurofilament light
73	64	hsa-miR-7-2-3p	NF-L, NEFL	neurofilament light
74	64	hsa-miR-5701	NF-L, NEFL	neurofilament light
75	64	hsa-miR-125b-2-3p	NF-L, NEFL	neurofilament light
76	63	hsa-miR-2276-3p	NF-L, NEFL	neurofilament light
77	63	hsa-let-7a-3p	NF-L, NEFL	neurofilament light
78	63	hsa-miR-98-3p	NF-L, NEFL	neurofilament light
79	63	hsa-miR-3934-3p	NF-L, NEFL	neurofilament light
80	63	hsa-let-7f-2-3p	NF-L, NEFL	neurofilament light
81	63	hsa-miR-4789-5p	NF-L, NEFL	neurofilament light
82	63	hsa-miR-5581-3p	NF-L, NEFL	neurofilament light
83	63	hsa-miR-1185-1-3p	NF-L, NEFL	neurofilament light
84	63	hsa-let-7b-3p	NF-L, NEFL	neurofilament light
85	63	hsa-miR-1185-2-3p	NF-L, NEFL	neurofilament light
86	63	hsa-let-7f-1-3p	NF-L, NEFL	neurofilament light
87	61	hsa-miR-6776-3p	NF-L, NEFL	neurofilament light
88	60	hsa-miR-7853-5p	NF-L, NEFL	neurofilament light
89	60	hsa-miR-105-5p	NF-L, NEFL	neurofilament light
90	60	hsa-miR-3672	NF-L, NEFL	neurofilament light
91	59	hsa-miR-3120-3p	NF-L, NEFL	neurofilament light
92	59	hsa-miR-3670	NF-L, NEFL	neurofilament light
93	59	hsa-miR-4795-3p	NF-L, NEFL	neurofilament light

94	59	hsa-miR-5579-5p	NF-L, NEFL	neurofilament light
95	59	hsa-miR-1182	NF-L, NEFL	neurofilament light
96	58	hsa-miR-577	NF-L, NEFL	neurofilament light
97	57	hsa-miR-491-5p	NF-L, NEFL	neurofilament light
98	57	hsa-miR-5571-5p	NF-L, NEFL	neurofilament light
99	56	hsa-miR-4798-5p	NF-L, NEFL	neurofilament light
100	55	hsa-miR-6792-5p	NF-L, NEFL	neurofilament light
101	55	hsa-miR-135a-3p	NF-L, NEFL	neurofilament light
102	55	hsa-miR-6128	NF-L, NEFL	neurofilament light
103	55	hsa-miR-411-3p	NF-L, NEFL	neurofilament light
104	55	hsa-miR-4774-5p	NF-L, NEFL	neurofilament light
105	55	hsa-miR-875-3p	NF-L, NEFL	neurofilament light
106	55	hsa-miR-379-3p	NF-L, NEFL	neurofilament light
107	55	hsa-miR-4443	NF-L, NEFL	neurofilament light
108	54	hsa-miR-4742-5p	NF-L, NEFL	neurofilament light
109	54	hsa-miR-589-3p	NF-L, NEFL	neurofilament light
110	53	hsa-miR-6811-5p	NF-L, NEFL	neurofilament light
111	53	hsa-miR-12119	NF-L, NEFL	neurofilament light
112	53	hsa-miR-6511b-5p	NF-L, NEFL	neurofilament light
113	53	hsa-miR-4328	NF-L, NEFL	neurofilament light
114	52	hsa-miR-1910-3p	NF-L, NEFL	neurofilament light
115	52	hsa-miR-1252-5p	NF-L, NEFL	neurofilament light
116	52	hsa-miR-582-3p	NF-L, NEFL	neurofilament light
117	52	hsa-miR-6511a-5p	NF-L, NEFL	neurofilament light
118	51	hsa-miR-7106-3p	NF-L, NEFL	neurofilament light
119	51	hsa-miR-3145-3p	NF-L, NEFL	neurofilament light
120	51	hsa-miR-3692-3p	NF-L, NEFL	neurofilament light
121	51	hsa-miR-4729	NF-L, NEFL	neurofilament light
122	50	hsa-miR-5589-3p	NF-L, NEFL	neurofilament light
123	50	hsa-miR-9-5p	NF-L, NEFL	neurofilament light
124	50	hsa-miR-203a-3p	NF-L, NEFL	neurofilament light
